# Baseline characteristics associated with early visual acuity gains after ranibizumab treatment for retinal vein occlusion

**DOI:** 10.1186/s12886-018-1012-y

**Published:** 2019-01-08

**Authors:** W. Lloyd Clark, Mimi Liu, John Kitchens, Pin-wen Wang, Zdenka Haskova

**Affiliations:** 1 Palmetto Retina Center, 124 Sunset Court, West Columbia, SC 29169 USA; 2 Colorado Retina Associates, Denver, CO USA; 3 Retina Associates of Kentucky, Lexington, KY USA; 4 Genentech, Inc., South San Francisco, CA USA

**Keywords:** Retinal vein occlusion, RVO, Branch retinal vein occlusion, BRVO, Central retinal vein occlusion, CRVO, Anti-VEGF, Ranibizumab

## Abstract

**Background:**

To identify baseline patient characteristics associated with early clinically significant visual acuity (VA) improvements within 3 months of treatment initiation in ranibizumab-treated patients with retinal vein occlusion (RVO) in the SHORE study.

**Methods:**

Post hoc analysis of baseline patient characteristics in the randomized, open-label, vision examiner–masked SHORE phase 4 study that compared monthly versus pro re nata dosing of ranibizumab in patients with branch and central RVO. Patients who enrolled in SHORE fulfilled eligibility criteria per protocol (*N* = 202). SHORE data were retrospectively analyzed to identify baseline patient characteristics associated with early clinically significant improvements in VA, defined as improvement to a Snellen equivalent of 20/40 or better vision (≥ 69 Early Treatment Diabetic Retinopathy Study [ETDRS] letters) or an increase in best-corrected VA (BCVA) of 15 or more ETDRS letters from baseline within 3 months of treatment initiation. Main outcome measures were BCVA gain of 15 or more ETDRS letters from baseline, Snellen equivalent of 20/40 or better vision, and baseline factors associated with early clinically significant improvement in BCVA.

**Results:**

The median time for patients to achieve a BCVA of 20/40 or better was 59 days and the median time for patients to gain 15 or more ETDRS letters was 63 days. Better baseline BCVA (> 50 ETDRS letters/Snellen equivalent ≥ 20/100), greater baseline total macular volume (> 9.99 mm^3^), and presence of subretinal fluid at baseline were all associated with early improvement to 20/40 or better vision (ETDRS equivalent ≥ 69 letters; *P* < .0001, *P* = .02, and *P* = .03, respectively).

**Conclusions:**

This retrospective analysis found that better BCVA, greater total macular volume, and presence of subretinal fluid at baseline were associated with more rapid vision gains. Clinicians may find these helpful when considering the likelihood of achieving early clinically significant VA improvements with ranibizumab in patients with RVO.

**Trial registration:**

ClinicalTrials.gov NCT01277302.

## Background

Retinal vein occlusion (RVO) is a common cause of retinal vascular disease [1] and both branch RVO (BRVO) and central RVO (CRVO) are associated with vision loss and decreased vision-related quality of life [2, 3]. Although RVO is most prevalent in older individuals, it also can occur in younger individuals [4], potentially compromising their ability to work and drive. The introduction of anti–vascular endothelial growth factor (VEGF) agents to treat macular edema secondary to RVO was an important advancement for the improvement of visual outcomes in patients with BRVO and CRVO [5]. Despite these advances, understanding and identifying the patients who will benefit the most and experience rapid vision improvement from anti-VEGF treatment remains an open and important question.

The randomized sham injection–controlled phase 3 BRAVO and CRUISE studies established the safety and efficacy of monthly treatment with the anti-VEGF agent ranibizumab for macular edema secondary to BRVO and CRVO, respectively [6–9]. The follow-up phase 4 SHORE study compared the efficacy of monthly and pro re nata (PRN) ranibizumab dosing on visual acuity (VA) maintenance in patients with BRVO and CRVO following disease activity stabilization [10]. The robust and clinically significant vision gains with ranibizumab during the initial 7-month-long monthly dosing period in SHORE were maintained over time with monthly and PRN dosing. Mean best-corrected VA (BCVA) gains from baseline at month 15 were 21.0 and 18.7 Early Treatment Diabetic Retinopathy Study (ETDRS) letters in the ranibizumab PRN and monthly arms, respectively [10].

The timing of patient responses to ranibizumab treatment in SHORE was heterogeneous. While the majority of patients met the prespecified disease stability criteria within 2 months of the end of the mandatory monthly loading phase (month 7 or month 8 of the study), a small number of patients failed to meet the disease stability criteria at any point during the study (*n* = 13) [10]. Although several baseline characteristics have been associated with an increased likelihood of VA improvements in patients with RVO treated with anti-VEGF agents in the limited literature available, the findings vary across studies and agents [11–13].

This retrospective exploratory analysis of SHORE data was designed to identify baseline predictors associated with early clinically significant VA improvement, defined as an increase in BCVA of 15 or more ETDRS letters from baseline or improvement to a Snellen equivalent of 20/40 or better vision, the threshold required to hold an unrestricted driver’s license across most of the United States. Understanding which baseline characteristics are associated with positive VA outcomes may help to inform both treatment expectations and approaches for patients with RVO.

## Methods

This was a post hoc analysis of data from the SHORE study, the methods of which have been published previously [10]. Briefly, SHORE was a 15-month, phase 4, multicenter, randomized, open-label study of patients with BRVO or CRVO (ClinicalTrials.gov identifier: NCT01277302; *N* = 202). The protocol was prospectively approved by the institutional review board at each study site, and all patients provided written informed consent. The study was conducted according to the International Conference on Harmonisation E6 Guideline for Good Clinical Practice and any national requirements. At enrollment, all study eyes had macular edema involving the foveal center due to BRVO or CRVO diagnosed within 12 months prior to screening. Key inclusion criteria for the study eye included ETDRS Protocol BCVA of 20/40–20/320 (Snellen equivalent). In SHORE, patients received 7 monthly ranibizumab 0.5 mg injections at months 0–6. Starting at the month 7 visit, each patient was assessed for potential randomization into the ranibizumab 0.5 mg monthly and ranibizumab 0.5 mg PRN treatment arms based on protocol-specified VA and anatomic disease stability criteria. Patients who did not meet the stability criteria were not randomized and continued to receive monthly ranibizumab 0.5 mg injections until study completion. Disease stability criteria and PRN re-treatment criteria have been previously reported by Campochiaro et al. [10].

The main objective of this exploratory analysis of SHORE was to identify baseline characteristics predictive of early visual improvements. The analyses were designed to determine the predictors of early vision gains in a wide group of patients who experienced meaningful improvements during the initial treatment period. Rather than focusing on a single preset time point, we examined the prevalence of the endpoints over time to define the timing of vision improvements across the population. To more closely mirror clinical practice, where patients present for their next office visits at variable times, we first determined the median time to clinically significant vision gains and then followed with the predictor analyses. We performed predictor analyses of the following visual outcomes: (1) time from baseline to first achievement of Snellen equivalent of 20/40 or better vision, and (2) time to first gain of 15 or more ETDRS letters from baseline. The baseline demographic and disease-associated characteristics that were evaluated for potential predictive value are summarized in Table 1. Baseline retinal nonperfusion status was not assessed for predictive value because more than 95% of patients had retinal nonperfusion at baseline, making a balanced sample size between present and absent groups impossible. Baseline predictor analyses of achievement of 20/40 or better vision excluded 22 (10.9%) patients who had 20/40 or better vision at baseline.

**Table 1:** Baseline Demographics and Patient Characteristics Assessed for Predictive Value of Early Visual Acuity Outcomes

Baseline predictors of early achievement of BCVA 20/40 or better (Snellen equivalent)	
BCVA (≤ 50, > 50) Subretinal fluid Total macular volume (≤ 9.99 mm^3^, > 9.99 mm^3^)	
Baseline predictors of early first BCVA gain of 15 or more ETDRS letters	
Sex	
Variables that did not have a predictive value on visual outcomes	
Age (≤ 60 years, > 60 years) Race BMI (< 30 kg/m^2^, ≥ 30 kg/m^2^) Disease type (BRVO/HRVO vs CRVO) Months since RVO diagnosis Smoking status Hypertension Diabetes Prior RVO therapies	Central subfield thickness (≤ 400 μm, > 400 μm) Edema Cystoid space Retinal hemorrhage Diffuse edema BCVA (≤ 35, 35–50, > 50 ETDRS letters) Treatment group DBP (< 80 mmHg, ≥ 80 mmHg) SBP (< 140 mmHg, ≥ 140 mmHg)

*BCVA* best-corrected visual acuity, *BMI* body mass index, *BRVO* branch retinal vein occlusion, *CRVO* central retinal vein occlusion, *DBP* diastolic blood pressure, *ETDRS* Early Treatment Diabetic Retinopathy Study, *HRVO* hemiretinal vein occlusion, *RVO* retinal vein occlusion, *SBP* systolic blood pressure

Time to first Snellen equivalent of 20/40 or better vision in the study eye (excluding eyes with 20/40 or better vision at baseline) and first gain of 15 or more ETDRS letters from baseline were analyzed using the Kaplan-Meier method. The log-rank test was used to compare the distribution of time to endpoints between baseline predictors. Median time to events was provided by baseline predictor groups. Baseline predictors evaluated were age, sex, race, body mass index, baseline disease type, months since RVO diagnosis, smoking status, hypertension, diabetes, prior therapies for RVO, central subfield thickness, subretinal fluid, total macular volume, edema, cystoid space, retinal hemorrhage, diffuse edema, BCVA, treatment, and diastolic and systolic blood pressures. Observed data were used for all endpoints without imputation for missing values.

## Results

Results from the primary analysis of SHORE have been reported previously [10]. Briefly, of the 202 patients in SHORE, 171 were randomized to monthly (*n* = 85) or PRN (*n* = 86) injections. In total, 31 patients were not randomized; 12 patients discontinued the study prior to month 7, 6 patients discontinued after month 7, and 13 patients completed the trial but did not meet the stability criteria during rolling randomization. Of those patients who met stability criteria, 136 (79.5%) were randomized by month 8 and only 10 (5.8%) were randomized after month 10. In the monthly and PRN injection groups, 80 (94.1%) patients and 82 (95.3%) patients completed the study, respectively. Mean (±SD) BCVA at baseline for all patients was 53.4 (±13.1) ETDRS letters, and 136 (67.3%) patients had a baseline BCVA of > 50 ETDRS letters (approximate Snellen equivalent: 20/100). The majority of study eyes were BRVO/hemiretinal vein occlusion (56.9%) versus CRVO (43.1%). Overall, 118 (58.4%) patients were male [10].

### Time to clinically significant vision gains

Rapid vision improvements were observed in patients treated with ranibizumab. At month 1, 101 of 200 (50.5%) patients had achieved 20/40 or better vision, an approximately 3- to 4-line gain of vision, on average, from the mean baseline vision of 20/100. Between months 4 and 15, 69.5–78.7% of all patients had 20/40 or better vision at each study visit. In the 180 patients who had vision worse than 20/40 at baseline, the median time to first reach 20/40 or better vision was 59 days. In total, 80.6% of the 180 patients achieved 20/40 or better vision at a minimum of 1 time point post baseline during the study period.

Similar results were found when evaluating the time to first gain of 15 or more ETDRS letters from baseline over the 15-month study period. After 1 month of ranibizumab therapy, 78 of 200 (39.0%) patients had gained 15 or more ETDRS letters from baseline. This improvement continued, as 61.1–71.1% of patients gained 15 or more ETDRS letters from baseline at each study visit between months 4 and 15. The median time for patients to first gain 15 or more ETDRS letters was 63 days. Overall, 171 of the 202 (84.7%) patients enrolled in the trial gained 15 or more ETDRS letters from baseline at a minimum of 1 time point post baseline during the study period.

### Baseline characteristics predictive of 20/40 or better vision

**Fig. 1:**
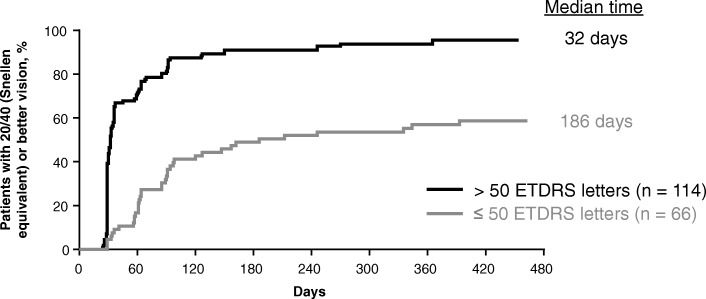
Time to achievement of 20/40 or better vision by baseline best-corrected visual acuity. Excludes 22 (10.9%) patients with 20/40 or better vision at baseline. ETDRS, Early Treatment Diabetic Retinopathy Study

**Fig. 2:**
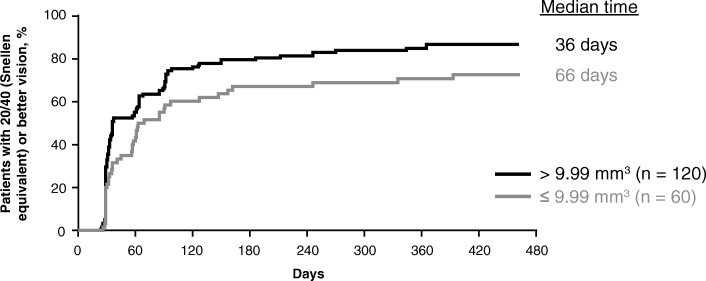
Time to achievement of 20/40 or better vision by baseline total macular volume. Excludes 22 (10.9%) patients with 20/40 or better vision at baseline; total macular volume was measured by the digital angiography reading center using spectral-domain optical coherence tomography

**Fig. 3:**
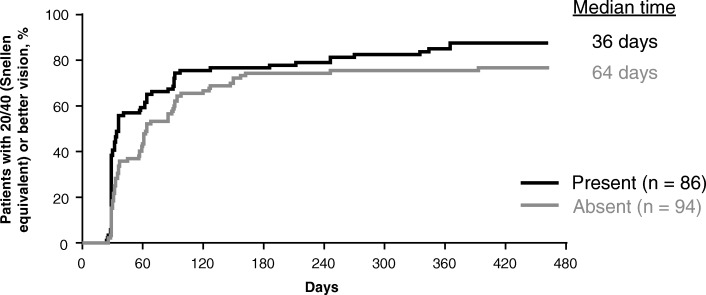
Time to achievement of 20/40 or better vision by baseline subretinal fluid status. Excludes 22 (10.9%) patients with 20/40 or better vision at baseline; subretinal fluid (presence or absence) by the digital angiography reading center was evaluated using spectral-domain optical coherence tomography

After examining more than 20 baseline predictors (Table 1), 3 characteristics emerged as predictive of early attainment of 20/40 or better vision within 3 months of treatment initiation: better baseline BCVA, greater baseline total macular volume, and presence of subretinal fluid at baseline. As seen in Fig. 1, patients with better baseline BCVA exhibited earlier improvement to 20/40 or better vision. The median time to first attain 20/40 or better vision was 32 days in patients with a baseline BCVA of 50 or more ETDRS letters (approximate Snellen equivalent: 20/100) compared with 186 days in patients with a baseline BCVA of less than or equal to 50 ETDRS letters (*P* < .0001). Early vision gains were also seen in patients with a greater baseline macular volume as measured on spectral-domain optical coherence tomography (Fig. 2). Patients with a macular volume of greater than 9.99 mm^3^ at baseline first achieved 20/40 or better vision in a median of 36 days compared with 66 days in patients with macular volume less than or equal to 9.99 mm^3^ at baseline (*P* = .02). Lastly, presence of subretinal fluid on spectral-domain optical coherence tomography at baseline was associated with earlier improvement to 20/40 or better vision (Fig. 3). Patients with subretinal fluid at baseline first reached 20/40 or better vision in a median of 36 days compared with 64 days in patients without subretinal fluid at baseline (*P* = .03).

### Baseline characteristics predictive of 15 or more ETDRS letter gains

Predictor analyses were also performed on the early attainment of vision gains of 15 or more ETDRS letters within 3 months of treatment initiation, and 2 characteristics of potential interest emerged. First, male sex was associated with an earlier first gain of 15 or more ETDRS letters. Male patients gained 15 or more ETDRS letters in a median of 58 days compared with 92 days for female patients (*P* = .03). The second characteristic of potential interest was worse BCVA at baseline. With a 43-day difference between the groups, patients with worse BCVA (≤ 50 ETDRS letters) at baseline exhibited a notably shorter median time to first gain of 15 or more ETDRS letters compared with patients with better BCVA (> 50 ETDRS letters) at baseline (49 vs 92 days, respectively). This association, however, was not statistically significant (*P* = .057).

## Discussion

RVO is a serious disease that may lead to irreversible vision loss and permanent disability. Prompt treatment is critical to avoid irreparable loss of vision. Here, we discuss correlations between baseline patient characteristics and early VA improvements to help guide physicians’ and patients’ expectations at the start of treatment.

In SHORE, ranibizumab treatment of patients with BRVO and CRVO resulted in rapid improvements in VA, with 39.0% of patients gaining 15 or more ETDRS letters from baseline after 1 injection, and 50.5% of patients achieving 20/40 or better vision after 1 injection. Rapid visual improvements are important both clinically and for patient quality of life. In clinical trials, the proportion of patients with vision gains of 15 or more ETDRS letters is a widely accepted clinically meaningful endpoint because gains of this magnitude represent a doubling of visual angle and correlate with the improvements in vision-related quality of life [14, 15]. Further, quick attainment of 20/40 or better vision can have a significant impact on a patient’s quality of life because it meets the VA threshold to obtain an unrestricted driver’s license throughout most of the United States [16]. In this subanalysis, the median times to first gain of 15 or more ETDRS letters and first attainment of 20/40 or better vision were approximately 2 months, showing the rapid efficacy of ranibizumab treatment in a large proportion of patients with RVO.

When examined in the context of other ranibizumab RVO trials, the clinically significant vision gains in SHORE occurred 50 or more days more quickly than in patients treated with ranibizumab 0.5 mg in BRAVO and CRUISE [14]. Although the reason for the difference in time to clinically significant vision gains remains unclear, several differences in the study populations may have played a role in the varied findings, including differences in time from diagnosis, baseline vision, and baseline disease severity.

To better understand which patients with RVO are more likely to show rapid clinically significant vision gains with ranibizumab treatment, this SHORE subanalysis examined a wide variety of baseline characteristics for their ability to predict achievement of early visual improvements within 3 months of treatment initiation. Of the more than 20 demographic, systemic, visual, and ocular anatomic baseline characteristics examined, the 3 factors predictive of earlier 20/40 or better vision were better BCVA (> 50 ETDRS letters; *P* < .0001), greater macular volume (*P* = .02), and presence of subretinal fluid at baseline (*P* = .03). Additional factors that were of potential interest for their association with earlier 15 or more ETDRS letter gains from baseline were male sex (*P* = .03) and worse baseline BCVA (≤ 50 ETDRS letters; *P* = .057).

The associations found between baseline BCVA status and early vision improvement were not surprising because baseline BCVA status played an important role in both VA metrics used in this analysis. For the early attainment of 20/40 or better vision metric, patients with better baseline BCVA (> 50 ETDRS letters) had an advantage because they were closer to the 20/40 threshold, allowing them to achieve 20/40 or better vision more quickly. In contrast, worse baseline vision (≤ 50 ETDRS letters) provided an advantage for the early attainment of 15 or more ETDRS letters from baseline because patients with worse baseline vision had greater room for improvement compared with patients with better baseline vision.

The predictive value of greater baseline macular volume and presence of subretinal fluid at baseline for early vision improvements is also anatomically plausible given the mechanism of action of ranibizumab and the pathophysiology of macular edema secondary to RVO. Patients with greater macular volume at baseline likely had increased disease severity caused by vascular permeability and fluid accumulation in the macula, factors known to disrupt vision. Thus, ranibizumab treatment allowed for rapid edema reduction and subsequent vision gains. Similarly, the rapid effects of ranibizumab on vascular permeability likely contributed to the early vision gains observed in ranibizumab-treated patients with subretinal fluid at baseline. Although anatomic features associated with fluid retention were associated with early vision gains, others such as retinal hemorrhage were not. These data indicate that vision loss due to edema was more rapidly and easily recoverable with ranibizumab treatment than vision loss due to retinal damage.

In this subanalysis of the SHORE study, male sex was associated with an earlier gain of 15 or more ETDRS letters. This finding is likely explained by the demographics of the cohort because the proportion of male patients with a Snellen equivalent of 20/200 or worse vision at baseline (22/118 [18.6%]) was nearly double that of female patients (8/84 [9.5%]; Table 2; *P* = .07). Further, male patients were slightly younger than female patients, and a higher proportion of male patients than female patients had the more visually debilitating diagnosis of CRVO. These differences could have created greater room for improvement in male patients, possibly resulting in faster VA improvement compared with female patients. Thus, it is unknown and perhaps unlikely that the finding that male sex was predictive of early visual improvements in patients with RVO treated with ranibizumab is generalizable to the overall population.

**Table 2:** Baseline Characteristics in Male Versus Female Patients

Characteristic	Male (*n* = 118)	Female (*n* = 84)
Mean age, years (SD)	64.0 (12.7)	69.5 (11.3)
Baseline occlusion type, n (%)		
BRVO/HRVO	59 (50.0)	56 (66.7)
CRVO	59 (50.0)	28 (33.3)
Mean BCVA, ETDRS letters (SD)	52.2 (14.0)	55.0 (11.6)
Patients with 20/200 or worse vision, n (%)	22 (18.6)	8 (9.5)
Mean CST, μm (SD)	539.0 (177.5)	508.6 (138.3)
Mean time since diagnosis of RVO, months (SD)	1.7 (2.1)	1.7 (1.7)
Prior therapies, n (%)		
Intravitreal steroids	7 (5.9)	2 (2.4)
Laser photocoagulation	1 (0.8)	1 (1.2)
Comorbidities, n (%)		
Open-angle glaucoma	10 (8.5)	7 (8.3)
Hypertension	70 (59.3)	61 (72.6)
Angina	4 (3.4)	3 (3.6)
Congestive heart failure	2 (1.7)	4 (4.8)
Diabetes mellitus	18 (15.3)	13 (15.5)

*BCVA* best-corrected visual acuity, *BRVO* branch retinal vein occlusion, *CRVO* central retinal vein occlusion, *CST* central subfield thickness, *ETDRS* Early Treatment Diabetic Retinopathy Study, *HRVO* hemiretinal vein occlusion, *RVO* retinal vein occlusion

Reports of specific baseline characteristics associated with VA improvements in anti-VEGF–treated patients with RVO vary throughout the literature, and include baseline BCVA, younger age, and time to treatment [11, 12]. Direct comparisons between analyses remain difficult due to the different parameters examined in each study. In particular, it is important to note that previous studies examined the effect of baseline characteristics on final VA, not the time to reach a clinically significant change in VA as examined in this SHORE subanalysis. In addition, clinical practice survey data indicate that the majority of patients with macular edema secondary to RVO are treated using a treat-and-extend or as-needed approach [17] instead of monthly, potentially limiting the generalizability of clinical trial findings.

This post hoc SHORE analysis of baseline predictors should be interpreted with caution because statistical analyses were not prespecified and this analysis may not have been sufficiently powered for all baseline predictor endpoints. In addition, because this was a post hoc analysis, baseline groups analyzed were not evenly distributed. For example, some of the predictor analyses had a distribution up to 2:1 between the compared groups (ie, total macular volume and baseline BCVA).

Despite these limitations, this SHORE analysis provides important hypotheses on the characteristics that may predict early treatment response in patients with macular edema secondary to BRVO and CRVO. The key baseline predictors of early VA improvement within 3 months of treatment initiation found in this analysis were baseline BCVA of more than 50 ETDRS letters, baseline macular volume greater than 9.99 mm^3^, and presence of subretinal fluid. A prospective study would be needed to confirm these hypotheses. Recognizing the baseline factors associated with early VA response to ranibizumab treatment in patients with BRVO and CRVO may help guide patients’ and physicians’ expectations at the start of therapy.

## Abbreviations

BCVA: Best-corrected visual acuity
BMI: Body mass index
BRVO: Branch retinal vein occlusion
CRVO: Central retinal vein occlusion
CST: Central subfield thickness
DBP: Diastolic blood pressure
ETDRS: Early Treatment Diabetic Retinopathy Study
HRVO: Hemiretinal vein occlusion
PRN: Pro re nata
RVO: Retinal vein occlusion
SBP: Systolic blood pressure
VA: Visual acuity
VEGF: Vascular endothelial growth factor
